# Preoperative platelet-lymphocyte ratio is superior to neutrophil-lymphocyte ratio as a prognostic factor for soft-tissue sarcoma

**DOI:** 10.1186/s12885-015-1654-6

**Published:** 2015-10-02

**Authors:** Yi Que, Haibo Qiu, Yuanfang Li, Yongming Chen, Wei Xiao, Zhiwei Zhou, Xing Zhang

**Affiliations:** 1 Department of Gastric and Pancreatic Surgery, Collaborative Innovation Center for Cancer Medicine, Sun Yat-sen University Cancer Center, 651 East Dongfeng Road, Guangzhou, 510060 China; 2 State Key Laboratory of Oncology in South China, Collaborative Innovation Center for Cancer Medicine, Sun Yat-sen University Cancer Center, 651 East Dongfeng Road, Guangzhou, 510060 China

**Keywords:** Soft tissue sarcoma, PLR, NLR, Prognosis, Overall survival

## Abstract

**Background:**

Inflammation can promote tumor growth, invasion, angiogenesis and even metastasis. Inflammatory markers have been identified as prognostic indicators in various malignances. This study compared the usefulness of platelet-lymphocyte ratio (PLR) with that of neutrophil-lymphocyte ratio (NLR) for predicting outcomes of patients who underwent radical resection for soft tissue sarcoma (STS).

**Methods:**

We included 222 STS patients in this retrospective study. Kaplan-Meier curves and multivariate Cox proportional models were used to calculate overall survival (OS) and disease free survival (DFS).

**Results:**

In univariate analysis, elevated PLR and NLR were both significantly associated with decreased OS. In multivariate analysis, PLR (HR: 2.60; 95 % CI: 1.17–5.74, *P* = 0.019) but not NLR was still identified as independent predictors of outcome. Median OS was 62 and 76 months for the high PLR and low PLR groups, respectively. High PLR and NLR were both significantly associated with shorter DFS in univariate analysis, with median DFS of 18 and 57 months in the high PLR and low PLR groups. In multivariate analysis, elevated PLR (HR: 1.77; 95 % CI: 1.05–2.97, *P* = 0.032) was also related to decreased DFS.

**Discussion:**

Our findings provide a new and valuable clue for diagnosing and monitoring STS. Prediction of disease progression is not only determined by the use of clinical or histopathological factors including tumor grade, tumor size, and tumor site but also by host-response factors such as performance status, weight loss, and systemic inflammatory response. They also significantly affect clinical outcomes. Thus, PLR can be used to enhance clinical prognostication. Furthermore, the PLR can be assessed from peripheral blood tests that are routinely available without any other complicated expenditure, thus providing lower cost and greater convenience for the prognostication.

**Conclusion:**

Elevated preoperative PLR as an independent prognostic factor is superior to NLR in predicting clinical outcome in patients with STS.

**Electronic supplementary material:**

The online version of this article (doi:10.1186/s12885-015-1654-6) contains supplementary material, which is available to authorized users.

## Background

Soft tissue sarcomas (STSs) account for less than 1 % of all cancers [1]. Primary treatments for STS include surgical resection with or without adjuvant radiation; however, the 5-year probability of local recurrence and metastasis remains high [2–4].

The prognosis of STS depends on clinical and histologic characteristics. Established prognostic and predictive factors are age at diagnosis, tumor size, tumor site, histologic grade, histologic subtype, tumor depth and margin status [5].

Several molecular biomarkers have also been associated with outcome in STS. For example, methylated RASSF1A was significantly related with the risk of death for STS patients [6]; high serum osteopontin is correlated with poor prognosis in STS [7]; Brownhill et al. have advocated use of the proliferation index (by detecting Ki-67) in a risk model of outcome for Ewing’s sarcoma [8]. However, this method is still under investigation and its clinical applications are limited by high costs.

The neoplasm microenvironment, as measured by a variety of blood parameters, significantly contributes to the development and progression of malignancies. For example, C-reactive protein, a non-specific blood biomarker of acute-phase inflammatory response, is often elevated in different cancer types [9–13]. Raised platelet counts predicts inferior survival in ovarian cancer, lung cancer, colon cancer, pancreatic cancer, and is potentially associated with mechanisms (such as increased thrombogenicity) that affect angiogenesis [14–17]. Additionally, patients with high neutrophil density reportedly have worse outcomes compared with those with low neutrophil density [18], whereas patients with high lymphocyte density apparently have better outcomes than those with low levels [19]. As NLR and PLR can be regarded as two representative indexes of systemic inflammation, we have used them to predict clinical outcome in patients with STSs.

To date, PLR has been identified as a reliable and easily accessible prognostic factor in ovarian cancer [20], colorectal cancer [21], breast cancer [22] and non-small-cell lung cancer [23]. NLR has also been shown to have prognostic value in various cancers [24, 25]. A meta-analysis of the prognostic value of blood NLR on clinical outcome in solid tumors showed that high NLR was associated with shorter survival [26]. Nevertheless, insufficient data exists for PLR versus NLR in STS. The aim of our study was to evaluate the effects of preoperative PLR and NLR on OS, and DFS in patients with soft-tissue sarcoma.

## Methods

### Subjects

We included 222 STS patients who underwent extensive and radical resection at the Sun Yat-sen University Cancer Center in Guangzhou, China, between 2000 and 2010 in this retrospective study. Written informed consent was obtained from each patient. Ethical approval was given by the medical ethics committee of Sun Yat-sen University Cancer Center IRB (reference number: B2014-03-20). All patients had confirmed STS, and none had received chemotherapy before collection of the blood count data. Patients were excluded if they presented with active infections, hematological disorders or malignancies, or autoimmune disorders, or if they were on steroids. Preoperative blood cell counts were obtained within 7 days before surgery by Sysmex XE-5000™ Automated Hematology System (Shanghai, China). Data, including clinical and histopathological parameters, were collected through database chart review. Disease staging was classified according to the American Joint Committee on Cancer (AJCC)7th Edition [27] and tumors were graded according to the French Federation of Cancer Centers Sarcoma Group grading system [28]. Adjuvant chemotherapy was administered in 39 patients (17.6 %), and adjuvant radiotherapy treatment in 65 patients (29.3 %). Doxorubicin-based combination chemotherapy regimens were mostly used in patients with postoperative chemotherapy. Patients with stage IV disease and a single resectable metastasis qualified for surgery; postoperative RT was administered to improve local control for patients with high-grade STS or positive surgical margins. Follow-up examinations were provided by the independent follow-up program department in Sun Yat-sen University at regular intervals (every 3 months in years 1–3, 6 months in years 4–5, and 12 months in years 6–15 after diagnosis).

### Statistical analysis

The primary end point of the study was OS, which was defined as the time from radical surgery to the date of death. The secondary end point of the study was DFS, which was calculated from the date of curative resection to the date of the tumor recurrence or distant metastasis. The DFS was censored at the time of death or at the last follow-up if the patient had remained disease-free by that time. Optimal cutoff values for the PLR and NLR were calculated by applying receiver operating curve (ROC) analysis. PLR was calculated as the absolute platelet count measured in × 10^9^/L, divided by the absolute lymphocyte count measured in × 10^9^/L. The NLR was calculated as the absolute neutrophil count measured in × 10^9^/L, divided by the absolute lymphocyte count measured in × 10^9^/L.

Associations between clinical and histopathological parameters with OS and DFS were analyzed using Kaplan-Meier curves and compared by the log-rank test. The chi-square (*Χ*^2^) test was used to analyze the relationship between PLR or NLR and clinicopathological parameters. Univariate and multivariate Cox-regression analyses were performed to determine effects of probable prognostic factors, including age, gender, performance status, diabetes mellitus, cardiopulmonary disease, smoking history, tumor depth, tumor site, tumor size, grade, adjuvant radiotherapy, adjuvant chemotherapy and AJCC stage on OS and DFS. Hazard ratios (HRs) estimated from the Cox analysis were reported as relative risks with corresponding 95 % confidence intervals(CIs). All analyses were performed using the SPSS statistical software package (SPSS statistics 17.0). *P* < 0.05 was considered as statistically significant.

## Results

### Patient characteristics and histologic subtype

The median age of the 222 patients with histologically confirmed STS who were included in the present study at surgery was 37 years (range, 5–78 years), and their median follow-up time was 74 months (range, 1–176 months [censored]). Patients were classified into different subtypes as shown in Table 1.

**Table 1:** Histologic type

	Number	Percent
Undifferentiated pleomorphic sarcoma/MFH	59	26.6
Fibrosarcoma	20	9.0
Dermatofibrosarcoma proberans	28	12.6
Well-differentiated liposarcoma	13	5.9
Myxoid liposarcoma	12	5.4
Pleomorphic liposarcoma	5	2.3
Leiomyosarcoma	13	5.9
Rhabdomyosarcoma	10	4.5
Synovial sarcoma	28	12.6
Epithelioid sarcoma	1	0.5
Angiosarcoma	8	3.6
Alveolar soft part sarcoma	5	2.3
MPNST	10	4.5
PNET	6	2.7
Malignant Triton Tumor	1	0.5
Mesenchymal chondrosarcoma	3	1.4

Patients’ mean blood values were as follows: platelet count: 252.02 ± 94.752; neutrophil count: 4.468 ± 2.543; lymphocyte count: 2.151 ± 0.707; PLR: 132.069 ± 80.600; and NLR: 2.407 ± 2.395. We used ROC analysis criteria to determine the optimal cutoffs as 133.915 (AUC: 0.640, 95 % CI: 0.541–0.739, *P* = 0.005), and 2.5 (AUC: 0.632, 95 % CI: 0.533–0.731, *P* = 0.009) for PLR and NLR, respectively.

### Relationships between PLR or NLR and other clinical characteristics

Elevated PLR was significantly associated with female sex, poor performance status, diabetes mellitus, smoking history, deep tumor depth, high tumor grade and large tumor size; Elevated NLR was significantly associated with poor performance status, deep tumor depth, high tumor grade, large tumor size, deep tumor site and high AJCC stage (Table 2).

**Table 2:** Clinical-pathological characteristics of soft tissue sarcoma patient

	Overall population *N* (%)	PLR			NLR		
		<133.915	≥133.915	*P*	<2.5	≥2.5	*P*
		*N* = 146	*N* = 76		*N* = 160	*N* = 62	
Age at operation(years)				0.091			0.067
<65	205(92.3)	138(94.5)	67(88.2)		151(94.4)	54(87.1)	
≥65	17(7.7)	8(5.5)	9(11.8)		9(5.6)	8(12.9)	
Gender				**0.02**			0.206
Female	96(43.2)	55(37.7)	41(53.9)		65(40.6)	31(50.0)	
Male	126(56.8)	91(62.3)	35(46.1)		95(59.4)	31(50.0)	
Performance status				**0.002**			**<0.001**
0 ~ 1	173(77.9)	123(84.2)	50(65.8)		137(85.6)	35(58.1)	
≥2	49(22.1)	23(15.8)	26(34.2)		23(14.4)	26(41.9)	
Diabetes mellitus				**0.013**			0.067
Yes	4(1.8)	0(0)	4(5.3)		1(0.6)	3(4.8)	
No	218(98.2)	146(100.0)	72(94.7)		159(99.4)	59(95.2)	
Cardiopulmonary disease				1.000			1.000
Yes	12(5.4)	8(5.5)	4(5.3)		9(5.6)	3(4.8)	
No	210(94.6)	138(94.5)	72(94.7)		151(94.4)	59(95.2)	
Ever smoked				**0.003**			0.300
Yes	34(15.3)	30(20.5)	4(5.3)		27(16.9)	7(11.3)	
No	188(84.7)	116(79.5)	72(94.7)		133(83.1)	55(88.7)	
Tumor depth				**0.024**			**0.001**
Superficial	87(39.2)	65(44.5)	22(28.9)		74(46.3)	13(21.0)	
Deep	135(60.8)	81(55.5)	54(71.1)		86(53.8)	49(79.0)	
Tumor grade				**0.028**			**0.047**
G1	65(29.3)	50(34.2)	15(19.7)		55(34.4)	10(16.1)	
G2	99(44.6)	65(44.5)	34(44.7)		67(41.9)	32(51.6)	
G3	36(16.2)	17(11.6)	19(25.0)		23(14.4)	13(21.0)	
Unknown	22(9.9)	14(9.6)	8(10.5)		15(9.4)	7(11.3)	
Tumor size				**0.005**			**<0.001**
<5 cm	105(47.3)	79(54.1)	26(34.2)		88(55.0)	17(27.4)	
≥5 cm	117(52.7)	67(45.9)	50(65.8)		72(45.0)	45(72.6)	
Tumor site				0.282			**0.002**
Upper extremity	21(9.5)	11(7.5)	10(13.2)		18(11.3)	3(4.8)	
Lower extremity	60(27.0)	41(28.1)	19(25.0)		46(28.8)	14(22.6)	
Thoracic/trunk	77(34.7)	54(37.0)	23(30.3)		62(38.8)	15(24.2)	
Intra-abdomina	35(15.8)	19(13.0)	16(21.1)		17(10.6)	18(29.0)	
Head-neck	29(13.1)	21(14.4)	8(10.5)		17(10.6)	12(19.4)	
AJCC stage				0.056			**0.002**
IA + IB	68(30.6)	52(35.6)	16(21.1)		57(35.6)	11(17.7)	
IIA + IIB	107(48.2)	68(46.6)	9(51.3)		77(48.1)	30(48.4)	
III + IV	34(15.3)	17(11.6)	17(22.4)		16(10.0)	18(29.0)	
Unknown	13(5.9)	9(6.2)	4(5.3)		10(6.3)	3(4.8)	

Bold print indicates statistical significance

### Prognostic significance of the clinical characteristics in STS

In univariate analysis, we found significant associations of performance status, tumor depth, tumor grade, tumor size, tumor site, AJCC stage, PLR and NLR with OS and DFS. In multivariate analysis, we observed significant associations of tumor site, AJCC stage and PLR, but not NLR with OS (Table 3). And significant associations remained among tumor depth, AJCC stage and PLR with DFS (Table 4). Multivariate analyses were performed based on age at surgery, gender, performance status, diabetes mellitus, cardiopulmonary disease, smoking history, tumor depth, tumor site, AJCC stage, adjuvant radiotherapy, adjuvant chemotherapy, PLR and NLR. The reason why factors such as tumor grade and tumor size were excluded is to eliminate the influence of statistical collinearity. Another multivariate analysis model including tumor grade and tumor size is available (Additional file 1: Table S1 and Additional file 2: Table S2).

**Table 3:** Univariate and multivariate Cox proportional analysis regarding overall survival

	Univariate analysis		Multivariate analysis	
Parameter	HR (95 % CI)	*P*-value	HR (95 % CI)	*P*-value
Age at operation(years)				
<65	1 (referent)	0.220	1 (referent)	0.219
≥65	1.70(0.73-4.00)		2.06(0.65-6.49)	
Gender				
Female	1 (referent)	0.772	1 (referent)	0.615
Male	1.09(0.62-1.89)		1.20(0.59-2.45)	
Performance status				
0 ~ 1	1 (referent)	**0.006**	1 (referent)	0.975
≥2	2.22 (1.26-3.93)		0.99 (0.48-2.03)	
Diabetes mellitus				
No	1 (referent)	0.943	1 (referent)	0.218
Yes	1.07(0.15-7.78)		0.27(0.03-2.18)	
Cardiopulmonary disease				
No	1 (referent)	0.344	1 (referent)	0.342
Yes	0.38(0.06-2.78)		0.33(0.03-3.27)	
Ever smoked				
No	1 (referent)	0.579	1 (referent)	0.273
Yes	1.23(0.60-2.52)		1.69 (0.66-4.32)	
Tumor depth				
Superficial	1 (referent)	**<0.001**	1 (referent)	0.096
Deep	6.09 (2.74-13.53)		2.41(0.85-6.77)	
Tumor grade				
G1	1 (referent)	**0.002**	NA	NA
G2	4.66(1.78-12.22)	**<0.001**		
G3	9.27(3.16-27.20)			
Tumor size				
<5 cm	1 (referent)	**0.001**	NA	NA
≥5 cm	2.87(1.55-5.32)			
Tumor site				
Trunk&extremity	1 (referent)	**<0.001**	1 (referent)	**0.002**
head/neck&intra-abdominal	4.48(2.57-7.81)		3.14 (1.52-6.48)	
AJCC stage				
IA + IB	1 (referent)	**0.001**	1 (referent)	**0.002**
IIA + IIB	5.13(1.97-13.37)	**<0.001**	3.92 (1.43-10.76)	**0.008**
III + IV	10.56 (3.71-30.08)		7.45(2.44-22.81)	
Adjuvant radiotherapy				
Yes	1 (referent)	0.798	1 (referent)	0.692
No	1.08 (0.60-1.95)		0.86(0.40-1.84)	
Adjuvant chemocherapy				
Yes	1 (referent)	0.320	1 (referent)	0.929
No	1.44 (0.70-2.97)		1.04(0.45-2.41)	
PLR				
<133.915z	1 (referent)	**0.002**	1 (referent)	**0.019**
≥133.915	2.49 (1.41-4.39)		2.60(1.17-5.74)	
NLR				
<2.5	1 (referent)	**<0.001**	1 (referent)	0.881
≥2.5	2.83 (1.61-4.99)		1.06(0.52-2.16)	

Bold print indicates statistical significance

**Table 4:** Univariate and multivariate Cox proportional analysis regarding disease-free-survival

	Univariate analysis		Multivariate analysis	
Parameter	HR (95 % CI)	*P*-value	HR (95 % CI)	*P*-value
Age at operation(years)				
<65	1 (referent)	0.362	1 (referent)	0.370
≥65	1.47 (0.64-3.37)		1.69(0.54-5.31)	
Gender				
Female	1 (referent)	0.436	1 (referent)	0.643
Male	0.85(0.56-1.29)		0.89(0.54-1.46)	
Performance status				
0 ~ 1	1 (referent)	**0.001**	1 (referent)	0.596
≥2	1.81(1.15-2.85)		1.16(0.68-1.97)	
Diabetes mellitus				
No	1 (referent)	**0.02**	1 (referent)	0.575
Yes	5.51(1.31-23.09)		0.66(0.16-2.78)	
Cardiopulmonary disease				
No	1 (referent)	0.510	1 (referent)	0.247
Yes	0.68(0.21-2.15)		2.52(0.53-12.06)	
Ever smoked				
No	1 (referent)	0.470	1 (referent)	0.064
Yes	1.23 (0.70-2.19)		1.95(0.96-3.96)	
Tumor depth				
Superficial	1 (referent)	**<0.001**	1 (referent)	**0.002**
Deep	4.07 (2.39-6.93)		2.841.47-5.49)	
Tumor grade				
G1	1 (referent)	**0.003**	NA	NA
G2	2.54 (1.37-4.71)	**<0.001**		
G3	6.71(3.43-13.12)			
Tumor size				
<5 cm	1 (referent)	**<0.001**	NA	NA
≥5 cm	2.22(1.43-3.45)			
Tumor site				
Trunk&extremity	1 (referent)	**<0.001**	1 (referent)	0.132
head/neck&intra-abdominal	2.26 (1.48-3.46)		1.49(0.89-2.52)	
AJCC stage				
IA + IB	1 (referent)	**0.001**	1 (referent)	**0.002**
IIA + IIB	2.72(1.51-4.89)	**<0.001**	1.85(0.98-3.50)	0.057
III + IV	5.37(2.72-10.61)		3.60(1.74-7.46)	
Adjuvant radiotherapy				
Yes	1 (referent)	0.216	1 (referent)	0.560
No	1.31(0.85-2.03)		1.17(0.70-1.95)	
Adjuvant chemocherapy				
Yes	1 (referent)	0.316	1 (referent)	0.753
No	1.30(0.78-2.19)		1.10(0.61-1.97)	
PLR				
<133.915	1 (referent)	**0.011**	1 (referent)	**0.032**
≥133.915	1.75(1.14-2.70)		1.77(1.05-2.97)	
NLR				
<2.5	1 (referent)	**0.018**	1 (referent)	0.516
≥2.5	1.71(1.10-2.66)		0.83(0.48-1.44)	

Bold print indicates statistical significance

### Prognostic significance of PLR and NLR in STS

**Fig. 1:**
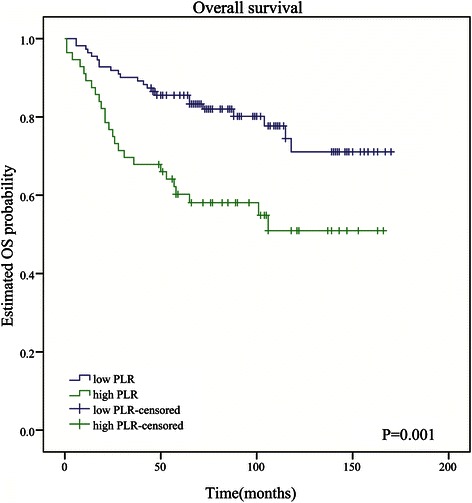
Kaplan-Meier curves for overall survival of patients with soft tissue sarcoma by low vs high platelet-lymphocyte ratio. PLR **≥** 133.915 is associated with poor survival (*P* = 0.001)

**Fig. 2:**
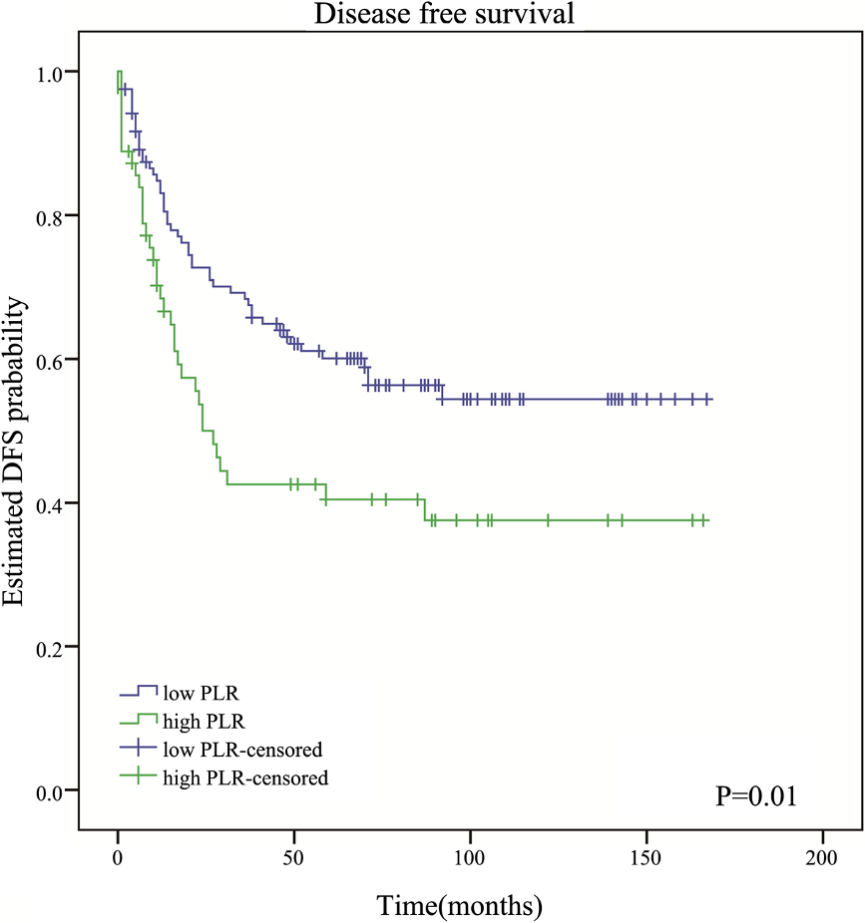
Kaplan-Meier curves for disease-free survival of patients with soft tissue sarcoma by low vs high platelet-lymphocyte ratio. PLR **≥** 133.915 is associated with poor survival (*P* = 0.01)

In univariate analysis, shorter OS was significantly associated with both high PLR (HR: 2.49; 95 % CI: 1.41–4.39; *P* = 0.002; Table 3; Fig. 1) and high NLR (HR: 2.83; 95 % CI: 1.61–4.99; *P* < 0.001; Table 3). In multivariate analysis, tumor site, AJCC stage, and PLR (HR: 2.60; 95 % CI: 1.17–5.74, *P* = 0.019) were still identified as independent prognostic factors (Table 3; Additional file 3: Table S3), but NLR was not (Table 3; Additional file 4: Table S4). Patients with high PLR had a median OS of 62 months, whereas those with low PLR had a median OS of 76 months. In univariate analyses, shorter DFS was associated with both high PLR (HR: 1.75; 95 % CI: 1.14–2.70, *P* = 0.011; Table 4; Fig. 2) and high NLR (HR: 1.71; 95 % CI: 1.10–2.66, *P* = 0.018; Table 4). However, elevated PLR (HR: 1.77; 95 % CI: 1.05–2.97, *P* = 0.032) but not NLR was independently associated with decreased DFS in multivariate analysis (Table 4). Patients with high PLR had a median DFS of 18 months, and those with low PLR had a median DFS of 57 months.

### Prognostic significance of PLR in different histologic types of STS

In subgroup analyses of the four major histologic types (undifferentiated [spindle cell and pleomorphic] sarcoma, fibrosarcoma, liposarcoma, and leiomyosarcoma), high PLR was associated with shorter OS in undifferentiated sarcoma in univariate analysis (HR: 3.50; 95 % CI: 1.21–10.11; *P* = 0.021; Table 5) and remained significant in multivariate analysis (HR: 3.91; 95 % CI: 1.02–14.99; *P* = 0.047; Table 5).

**Table 5:** Association of prognostic factors and PLR with overall survival in specific histologic tumor types

	Univariate analysis		Multivariate analysis	
Parameter	HR (95 % CI)	*P*-value	HR (95 % CI)	*P*-value
Undifferentiated(spindle cell and pleomorphic) sarcoma				
	1 (referent)	**0.021**	1 (referent)	**0.047**
	3.50(1.21-10.11)		3.91(1.02-14.99)	
Fibrosarcoma				
	1 (referent)	0.160	1 (referent)	0.157
	2.81(0.67-11.81)		3.16(0.64-15.59)	
Liposarcoma				
	1 (referent)	0.177	1 (referent)	NA
	5.22(0.47-57.67)		NA	
Leiomyosarcoma				
	1 (referent)	0.425	1 (referent)	NA
	2.08(0.34-12.62)		NA	

Bold print indicates statistical significance

## Discussion

Our present study showed that high preoperative PLR is independently associated with survival in patients who underwent extensive radical surgery.

Accumulating evidence has shown that platelets can support various steps of cancer development and tumor progression by promoting cancer cell proliferation, tumor angiogenesis and metastasis. In addition to their function in hemostasis, platelets are also involved in inflammatory disease and cancer [29]. Platelets reportedly have a stimulatory effect on ovarian cancer cell proliferation via the transforming growth factor (TGF)-β [30]. They have also been shown in vitro to inhibit apoptosis and reverse cell-cycle arrest induced by chemotherapeutic agents (such as 5-fluorouracil and paclitaxel) and enhance DNA repair in cancer cells [31]. Secondly, as tumor growth seems to depend on the formation of new blood vessels [32], platelets, which carry a variety of proangiogenic factors, affect regulation of cancer angiogenesis. Interestingly, cancer cells were also suggested to induce release of vascular endothelial growth factor from platelets, resulting in angiogenesis [33]. Platelets have been linked to tumor metastasis [34, 35] with underlying mechanisms that include attenuating the ability of natural killer cells to shield circulating cancer cells against the immune system [36] and inducing epithelial–mesenchymal transition [37].

As with platelets, lymphocytes are a significant blood parameter related to immune surveillance. Thus, high lymphocytic infiltrate is associated with improved survival and superior response to systemic therapy [38, 39] whereas a low peripheral blood lymphocyte counts are related to poor cancer prognoses [40, 41].

A combined index of platelet and lymphocyte counts has been investigated as prognostic factor for some cancers. Recently, a meta-analysis, comprising 12,754 patients, of the association of blood PLR and overall survival in solid tumors concluded that high PLR was independently associated with shorter OS in various solid tumors [42]. Asher et al. reported that high preoperative PLR was associated with poor survival in ovarian cancer [20]; and Krenn-Pilko et al. found that preoperative PLR as an independent prognostic marker for survival in breast cancer patients [22]. Szkandera et al. evaluated the prognostic significance of PLR in STS patients and found statistically significant associations in univariate, but not multivariate analyses, and that high preoperative NLR was an independent prognostic factor in multivariate analysis [43, 44], which differed from our results. However, their studies used different cancer populations, different NLR and PLR cut-off values, and patient cohorts of a different median age from our study, which might hinder the comparability of their results with ours. Moreover, these inflammatory factors may be affected by potential confounding factors, including smoking history, performance status and co-morbidities. Thus, the significance of inflammatory markers in STS requires further evaluation.

Findings that PLR is superior to NLR in predicting clinical outcomes vary in different studies that address different cancers. Our findings are consistent with some prior studies [20, 45], but not others [46, 47]. As we have mentioned, differences in race [48] or cutoff values may affect the results. Racial variations are known to affect cutoff values. For example, Caucasians have higher peripheral blood neutrophil counts and lower lymphocyte counts than do Asians [49]; NLR ≥ 5 was considered high in reports on Caucasian patients [50–52], whereas some studies on Asian patients used NLR >3 and >4 as cutoff points [53, 54]. For PLR, some reports used 150 or 300 as cutoff points [21, 53], some studies identified the ideal cutoff value by applying ROC curve and the cutoff points [22, 23].

Our findings provide a new and valuable clue for diagnosing and monitoring STS. Prediction of disease progression is not only determined by the use of clinical or histopathological factors including tumor grade, tumor size, and tumor site but also by host-response factors [55], such as performance status, weight loss, and systemic inflammatory response [56]. They also significantly affect clinical outcomes [57]. Thus, PLR can be used to enhance clinical prognostication. Furthermore, the PLR can be assessed from peripheral blood tests that are routinely available without any other complicated expenditure, thus providing lower cost and greater convenience for the prognostication.

Nevertheless, this study has some limitations, namely its retrospective research design. The unavailability of data regarding cancer-specific survival is another limitation. Choi et al. assessed multiple preoperative systemic inflammatory serum markers and predicted an association between high inflammatory status and shorter disease-specific survival in STS [58]. They showed that inflammatory marker values were significantly associated with histologic grade. Furthermore, the presence of multiple elevated markers was the most significant predictor of disease-specific survival. As NLR may vary by race [59], the fact that > 95 % of our patients were Asians is another limitation. Additionally, thrombocytosis and lymphocytopenia could have other causes, including bacterial infections, connective tissue disorders, intense physical exercise, severe stress. Nevertheless, the association of poor clinical outcome with high PLR in our results has not been challenged, considering these limitations.

## Conclusion

Our study indicates that PLR is an independent prognostic factor for survival of STS. Validation studies or large-scale prospective studies are warranted to verify our findings.

## Additional files

Additional file 1: Table S1. Multivariate analysis model 2 predicting overall survival using platelet-lymphocyte- ratio (PLR). (XLSX 11 kb)
Additional file 2: Table S2. Multivariate analysis model 2 predicting overall survival using neutrophil-lymphocyte-ratio (NLR). (XLSX 11 kb)
Additional file 3: Table S3. Multivariate analysis model predicting overall survival using platelet-lymphocyte- ratio (PLR). (XLSX 11 kb)
Additional file 4: Table S4. Multivariate analysis model predicting overall survival using neutrophil-lymphocyte-ratio (NLR). (XLSX 11 kb)

## Abbreviations

AJCC: American Joint Committee on Cancer
CI: Confidence interval
DFS: Disease-free survival
HR: Hazard ratio
NLR: Neutrophil-lymphocyte ratio
OS: Overall survival
PLR: Platelet-lymphocyte ratio
ROC: Receiver operating curve
STS: Soft tissue sarcoma
MFH: Malignant fibrous histiocytoma
MPNST: Malignant peripheral nerve sheath tumor
PNET: Primitive neuroectodermal tumor

## References

[1] Siegel R, Ward E, Brawley O, Jemal A (2011). Cancer statistics, 2011: the impact of eliminating socioeconomic and racial disparities on premature cancer deaths. CA Cancer J Clin.

[2] Daigeler A, Zmarsly I, Hirsch T, Goertz O, Steinau HU, Lehnhardt M (2014). Long-term outcome after local recurrence of soft tissue sarcoma: a retrospective analysis of factors predictive of survival in 135 patients with locally recurrent soft tissue sarcoma. Br J Cancer.

[3] Kang S, Kim HS, Kim S, Kim W, Han I (2014). Post-metastasis survival in extremity soft tissue sarcoma: a recursive partitioning analysis of prognostic factors. Eur J Cancer.

[4] Weitz J, Antonescu CR, Brennan MF (2003). Localized extremity soft tissue sarcoma: improved knowledge with unchanged survival over time. J Clin Oncol.

[5] Kattan MW, Leung DH, Brennan MF (2002). Postoperative nomogram for 12-year sarcoma-specific death. J Clin Oncol.

[6] Seidel C, Bartel F, Rastetter M, Bluemke K, Wurl P, Taubert H (2005). Alterations of cancer-related genes in soft tissue sarcomas: hypermethylation of RASSF1A is frequently detected in leiomyosarcoma and associated with poor prognosis in sarcoma. Int. J. Cancer.

[7] Bache M, Kappler M, Wichmann H, Rot S, Hahnel A, Greither T (2010). Elevated tumor and serum levels of the hypoxia-associated protein osteopontin are associated with prognosis for soft tissue sarcoma patients. BMC Cancer.

[8] Brownhill S, Cohen D, Burchill S (2014). Proliferation index: a continuous model to predict prognosis in patients with tumours of the Ewing's sarcoma family. PLoS One.

[9] Guillem P, Triboulet JP (2005). Elevated serum levels of C-reactive protein are indicative of a poor prognosis in patients with esophageal cancer. Dis Esophagus.

[10] Schmid M, Schneitter A, Hinterberger S, Seeber J, Reinthaller A, Hefler L (2007). Association of elevated C-reactive protein levels with an impaired prognosis in patients with surgically treated endometrial cancer. Obstet Gynecol.

[11] Allin KH, Nordestgaard BG, Flyger H, Bojesen SE (2011). Elevated pre-treatment levels of plasma C-reactive protein are associated with poor prognosis after breast cancer: a cohort study. Breast Cancer Res.

[12] Polterauer S, Grimm C, Zeillinger R, Heinze G, Tempfer C, Reinthaller A (2011). Association of C-reactive protein (CRP) gene polymorphisms, serum CRP levels and cervical cancer prognosis. Anticancer Res.

[13] Kersten C, Louhimo J, Algars A, Lahdesmaki A, Cvancerova M, Stenstedt K (2013). Increased C-reactive protein implies a poorer stage-specific prognosis in colon cancer. Acta Oncol.

[14] Gonzalez Barcala FJ, Garcia Prim JM, Moldes Rodriguez M, Alvarez Fernandez J, Rey Rey MJ, Pose Reino A (2010). Platelet count: association with prognosis in lung cancer. Med Oncol.

[15] Lin MS, Huang JX, Zhu J, Shen HZ (2012). Elevation of platelet count in patients with colorectal cancer predicts tendency to metastases and poor prognosis. Hepatogastroenterology.

[16] Qiu J, Yu Y, Fu Y, Ye F, Xie X, Lu W (2012). Preoperative plasma fibrinogen, platelet count and prognosis in epithelial ovarian cancer. J Obstet Gynaecol Res.

[17] Dominguez I, Crippa S, Thayer SP, Hung YP, Ferrone CR, Warshaw AL (2008). Preoperative platelet count and survival prognosis in resected pancreatic ductal adenocarcinoma. World J Surg.

[18] Teramukai S, Kitano T, Kishida Y, Kawahara M, Kubota K, Komuta K (2009). Pretreatment neutrophil count as an independent prognostic factor in advanced non-small-cell lung cancer: an analysis of Japan Multinational Trial Organisation LC00-03. Eur J Cancer.

[19] Clark EJ, Connor S, Taylor MA, Madhavan KK, Garden OJ, Parks RW (2007). Preoperative lymphocyte count as a prognostic factor in resected pancreatic ductal adenocarcinoma. HPB (Oxford).

[20] Asher V, Lee J, Innamaa A, Bali A (2011). Preoperative platelet lymphocyte ratio as an independent prognostic marker in ovarian cancer. Clin Transl Oncol.

[21] Kwon HC, Kim SH, Oh SY, Lee S, Lee JH, Choi HJ (2012). Clinical significance of preoperative neutrophil-lymphocyte versus platelet-lymphocyte ratio in patients with operable colorectal cancer. Biomarkers.

[22] Krenn-Pilko S, Langsenlehner U, Thurner EM, Stojakovic T, Pichler M, Gerger A (2014). The elevated preoperative platelet-to-lymphocyte ratio predicts poor prognosis in breast cancer patients. Br J Cancer.

[23] Liu H, Wu Y, Wang Z, Yao Y, Chen F, Zhang H (2013). Pretreatment platelet-to-lymphocyte ratio (PLR) as a predictor of response to first-line platinum-based chemotherapy and prognosis for patients with non-small cell lung cancer. J Thorac Dis.

[24] Guthrie GJ, Charles KA, Roxburgh CS, Horgan PG, McMillan DC, Clarke SJ (2013). The systemic inflammation-based neutrophil-lymphocyte ratio: experience in patients with cancer. Crit Rev Oncol Hematol.

[25] Malietzis G, Giacometti M, Kennedy RH, Athanasiou T, Aziz O, Jenkins JT (2014). The emerging role of neutrophil to lymphocyte ratio in determining colorectal cancer treatment outcomes: a systematic review and meta-analysis. Ann Surg Oncol.

[26] Templeton AJ, McNamara MG, Seruga B, Vera-Badillo FE, Aneja P, Ocana A (2014). Prognostic role of neutrophil-to-lymphocyte ratio in solid tumors: a systematic review and meta-analysis. Journal of the National Cancer Institute.

[27] Edge SB, Compton CC (2010). The American Joint Committee on Cancer: the 7th edition of the AJCC cancer staging manual and the future of TNM. Ann Surg Oncol.

[28] Neuville A, Chibon F, Coindre JM (2014). Grading of soft tissue sarcomas: from histological to molecular assessment. Pathology.

[29] Leslie M (2010). Cell biology. Beyond clotting: the powers of platelets. Science.

[30] Cho MS, Bottsford-Miller J, Vasquez HG, Stone R, Zand B, Kroll MH (2012). Platelets increase the proliferation of ovarian cancer cells. Blood.

[31] Radziwon-Balicka A, Medina C, O'Driscoll L, Treumann A, Bazou D, Inkielewicz-Stepniak I (2012). Platelets increase survival of adenocarcinoma cells challenged with anticancer drugs: mechanisms and implications for chemoresistance. Br J Pharmacol.

[32] Kerbel RS (2008). Tumor angiogenesis. N Engl J Med.

[33] Battinelli EM, Markens BA, Italiano JE (2011). Release of angiogenesis regulatory proteins from platelet alpha granules: modulation of physiologic and pathologic angiogenesis. Blood.

[34] Gay LJ, Felding-Habermann B (2011). Contribution of platelets to tumour metastasis. Nat Rev Cancer.

[35] Gasic GJ, Gasic TB, Stewart CC (1968). Antimetastatic effects associated with platelet reduction. Proc Natl Acad Sci U S A.

[36] Nieswandt B, Hafner M, Echtenacher B, Mannel DN (1999). Lysis of tumor cells by natural killer cells in mice is impeded by platelets. Cancer Res.

[37] Labelle M, Begum S, Hynes RO (2011). Direct signaling between platelets and cancer cells induces an epithelial-mesenchymal-like transition and promotes metastasis. Cancer Cell.

[38] Mahmoud SM, Paish EC, Powe DG, Macmillan RD, Grainge MJ, Lee AH (2011). Tumor-infiltrating CD8+ lymphocytes predict clinical outcome in breast cancer. J Clin Oncol.

[39] Seo AN, Lee HJ, Kim EJ, Kim HJ, Jang MH, Lee HE (2013). Tumour-infiltrating CD8+ lymphocytes as an independent predictive factor for pathological complete response to primary systemic therapy in breast cancer. Br J Cancer.

[40] Fogar P, Sperti C, Basso D, Sanzari MC, Greco E, Davoli C (2006). Decreased total lymphocyte counts in pancreatic cancer: an index of adverse outcome. Pancreas.

[41] Ray-Coquard I, Cropet C, Van Glabbeke M, Sebban C, Le Cesne A, Judson I (2009). Lymphopenia as a prognostic factor for overall survival in advanced carcinomas, sarcomas, and lymphomas. Cancer Res.

[42] Templeton AJ, Ace O, McNamara MG, Al-Mubarak M, Vera-Badillo FE, Hermanns T (2014). Prognostic role of platelet to lymphocyte ratio in solid tumors: a systematic review and meta-analysis. Cancer Epidemiol. Biomarkers Prev..

[43] Szkandera J, Gerger A, Liegl-Atzwanger B, Absenger G, Stotz M, Friesenbichler J (2014). The lymphocyte/monocyte ratio predicts poor clinical outcome and improves the predictive accuracy in patients with soft tissue sarcomas. Int J Cancer.

[44] Szkandera J, Absenger G, Liegl-Atzwanger B, Pichler M, Stotz M, Samonigg H (2013). Elevated preoperative neutrophil/lymphocyte ratio is associated with poor prognosis in soft-tissue sarcoma patients. Br J Cancer.

[45] Smith RA, Bosonnet L, Raraty M, Sutton R, Neoptolemos JP, Campbell F (2009). Preoperative platelet-lymphocyte ratio is an independent significant prognostic marker in resected pancreatic ductal adenocarcinoma. Am J Surg.

[46] Azab B, Shah N, Radbel J, Tan P, Bhatt V, Vonfrolio S (2013). Pretreatment neutrophil/lymphocyte ratio is superior to platelet/lymphocyte ratio as a predictor of long-term mortality in breast cancer patients. Med Oncol.

[47] Azab B, Mohammad F, Shah N, Vonfrolio S, Lu W, Kedia S (2014). The value of the pretreatment neutrophil lymphocyte ratio vs. platelet lymphocyte ratio in predicting the long-term survival in colorectal cancer. Cancer Biomark.

[48] Giri S, Shrestha R, Pathak R, Bhatt VR (2015). Racial Differences in the Overall Survival of Hairy Cell Leukemia in the United States: A Population-Based Analysis of the Surveillance, Epidemiology, and End Results Database. Clin Lymphoma Myeloma Leuk.

[49] Bain B, Seed M, Godsland I (1984). Normal values for peripheral blood white cell counts in women of four different ethnic origins. J Clin Pathol.

[50] Halazun KJ, Hardy MA, Rana AA, Woodland DC, Luyten EJ, Mahadev S (2009). Negative impact of neutrophil-lymphocyte ratio on outcome after liver transplantation for hepatocellular carcinoma. Ann Surg.

[51] Kishi Y, Kopetz S, Chun YS, Palavecino M, Abdalla EK, Vauthey JN (2009). Blood neutrophil-to-lymphocyte ratio predicts survival in patients with colorectal liver metastases treated with systemic chemotherapy. Ann Surg Oncol.

[52] Idowu OK, Ding Q, Taktak AF, Chandrasekar CR, Yin Q (2012). Clinical implication of pretreatment neutrophil to lymphocyte ratio in soft tissue sarcoma. Biomarkers.

[53] He W, Yin C, Guo G, Jiang C, Wang F, Qiu H (2013). Initial neutrophil lymphocyte ratio is superior to platelet lymphocyte ratio as an adverse prognostic and predictive factor in metastatic colorectal cancer. Med Oncol.

[54] Ding PR, An X, Zhang RX, Fang YJ, Li LR, Chen G (2010). Elevated preoperative neutrophil to lymphocyte ratio predicts risk of recurrence following curative resection for stage IIA colon cancer. Int J Colorectal Dis.

[55] MacDonald N (2007). Cancer cachexia and targeting chronic inflammation: a unified approach to cancer treatment and palliative/supportive care. J Support Oncol.

[56] Graf W, Bergstrom R, Pahlman L, Glimelius B (1994). Appraisal of a model for prediction of prognosis in advanced colorectal cancer. Eur J Cancer.

[57] Maltoni M, Caraceni A, Brunelli C, Broeckaert B, Christakis N, Eychmueller S (2005). Prognostic factors in advanced cancer patients: evidence-based clinical recommendations--a study by the Steering Committee of the European Association for Palliative Care. J Clin Oncol.

[58] Choi ES, Kim HS, Han I (2014). Elevated preoperative systemic inflammatory markers predict poor outcome in localized soft tissue sarcoma. Ann Surg Oncol.

[59] Azab B, Camacho-Rivera M, Taioli E (2014). Average values and racial differences of neutrophil lymphocyte ratio among a nationally representative sample of United States subjects. PLoS One.

